# Patient Safety Communication Priorities Among New Graduate Nurses: A Cross-Sectional Study

**DOI:** 10.3390/healthcare14131933

**Published:** 2026-07-01

**Authors:** Haena Jang

**Affiliations:** College of Nursing, Dong-A University, Busan 49201, Republic of Korea; hnjang@dau.ac.kr

**Keywords:** patient safety, communication, importance-performance analysis, needs assessment, education, nursing, new graduate nurses

## Abstract

**Highlights:**

**What are the main findings?**

- The Importance-Performance Analysis (IPA) revealed that new graduate nurses’ confidence levels were significantly lower than their perceived importance scores across all patient safety communication items.
- While effective verbal and non-verbal communication (C1) was identified as a strength (Quadrant I), communication in high-risk situations (C2) emerged as an area requiring focused educational attention (Quadrant II).

**What are the implications of the main findings?**

- The gap between importance and confidence scores suggests the need to strengthen undergraduate curricula to better enhance new graduate nurses’ confidence in patient safety communication.
- Nursing programs should integrate targeted, scenario-based communication training focused on high-risk clinical situations to facilitate a smoother transition from graduation to clinical practice.

**Abstract:**

**Background/Objectives:** This study aimed to identify educational priorities for patient safety communication among new graduate nurses using Importance-Performance Analysis (IPA). **Methods:** This cross-sectional, descriptive, secondary analysis involved 142 new graduates from four colleges in South Korea. Participants’ communication competencies were evaluated using a tool based on the Canadian Patient Safety Institute (CPSI) framework. IPA was used to prioritize four patient safety communication competencies and 28 items by comparing perceived importance with confidence levels. **Results:** The perceived importance of patient safety communication was significantly higher than confidence across all domains (*p* < 0.001). Based on the IPA matrix, verbal and non-verbal communication (importance: 4.37, confidence: 3.77) was positioned in Quadrant I (‘keep up the good work’). Communication in high-risk situations (importance: 4.31, confidence: 3.36) was classified in Quadrant II (‘concentrate here’). Written communication (importance: 4.18, confidence: 3.46) and communication technologies (importance: 4.20, confidence: 3.42) were classified in Quadrant III (‘low priority’). **Conclusions:** To bridge the gap between importance and confidence, it is crucial to identify specific high-risk clinical scenarios and develop educational programs that emphasize practical communication skills. These educational efforts should be strengthened in both undergraduate nursing curricula and nurse residency programs to enhance the clinical readiness of new nurses.

## 1. Introduction

Effective communication among healthcare workers is a fundamental requirement for ensuring patient safety and quality of care [1,2]. Failure to communicate effectively, including incomplete, inaccurate, or omitted critical information, has been identified as a major cause of adverse events, including sentinel events [3].

The Canadian Patient Safety Institute (CPSI), a leading organization in the field of patient safety, emphasized communication as a distinct domain among six safety competencies. Healthcare professionals are expected to develop verbal and non-verbal communication skills (e.g., employing active listening and demonstrating empathy), which are key competencies for patient safety communication, and to communicate effectively in high-risk situations that threaten safety, such as transitions of care or situations requiring escalation of clinical concerns. In addition, they should use clear documentation (e.g., detailed charting to avoid misinterpretation) and communication technology (e.g., utilizing electronic health records and telephone read-backs) to support safe care [4].

Among healthcare professionals, nurses play a key role in systematically and routinely evaluating and monitoring patients’ health status and responses to prevent harm [5,6]. Accordingly, nursing students, who are future nurses, should be taught to communicate effectively with other healthcare personnel during undergraduate education to improve patient health outcomes [6,7,8]. Communication competency is therefore considered a central goal by nursing education institutions such as the American Association of Colleges of Nursing [6] and Quality and Safety Education for Nurses [9,10]. In Korea, communication competency was identified as the top priority for improvement in nursing curricula [7], and safety was added as a key program outcome in the fourth nursing education accreditation evaluation [11].

However, despite ongoing educational efforts, the extent and approaches of patient safety education vary across nursing programs [8,12]. Previous research has also shown that new graduate nurses often experience difficulties and limited confidence in communicating with other healthcare professionals, such as physicians, during their transition to clinical practice [13]. In this context, while systematic evaluation of patient safety communication competencies is needed, few studies have comprehensively examined these competencies among new graduate nurses [14]—particularly by considering both their perceived importance and confidence levels. Therefore, a systematic educational needs analysis is necessary to provide targeted educational support, enabling new graduate nurses to communicate more effectively for patient safety.

Importance-performance analysis (IPA) is a well-established strategy for systematically identifying priorities for improvement. Originally designed to evaluate consumer perceptions of products in the automobile industry and devise marketing strategies, IPA utilizes a matrix with four quadrants based on importance (*y*-axis) and performance (*x*-axis): Quadrant Ι (keep up the good work), Quadrant II (concentrate here), Quadrant III (low priority), and Quadrant IV (possible overkill) [15]. This framework enables researchers to identify areas requiring immediate improvement while distinguishing competencies that should be maintained or given lower priority. IPA is now widely recognized as a systematic tool for prioritizing resource allocation in various fields, including education and healthcare. In healthcare education, IPA has been used to identify gaps between perceived importance and performance-related indicators in competencies related to clinical practice, patient safety, and professional training, thereby informing targeted educational interventions and resource allocation. Within patient safety nursing, this approach has been applied to identify work priorities for dedicated safety personnel [16] and to evaluate specific nursing activities [17].

Therefore, this study aimed to identify educational priorities for patient safety communication among new graduate nurses using IPA. Specifically, it assessed the perceived importance and confidence levels across four core communication competencies based on the CPSI framework [4] and explored priority areas for curriculum enhancement and clinical transition support. By identifying these priorities, the study provides empirical insights for strengthening nursing communication education programs to enhance patient safety.

## 2. Materials and Methods

### 2.1. Design and Participants

This study used a cross-sectional, descriptive, secondary analysis design. The original data were collected from a convenience sample of nursing graduates from four nursing colleges in South Korea. Data collection was conducted from March to April 2020, via an online survey platform (Qualtrics, Provo, UT, USA). To recruit participants, a recruitment notice containing a detailed description of the study was posted on online community boards for nursing students. A link directing potential participants to the informed consent form and the survey was provided at the bottom of the notice, allowing them to access and participate in the study voluntarily.

The inclusion criteria were: (1) individuals who graduated from a nursing program in 2020 and (2) those who had graduated within the past three months at the time of the survey. The exclusion criteria were individuals who declined to provide or subsequently withdrew their voluntary informed consent to participate in the study.

Secondary analysis refers to the process of re-analyzing data after the emergence of new research questions based on existing data or better statistical techniques [18]. The original survey included parallel ratings of perceived importance and confidence for each patient safety communication competency, which enabled the present secondary analysis to address educational priorities using IPA. Distinct from the primary study, which focused on developing and validating the scale, the present study reanalyzed the existing importance and confidence ratings to examine gaps between perceived importance and confidence and identify educational priorities.

In the original data collection, 239 graduates initiated the survey, and 142 completed it (dropout rate: 40.6%). A power analysis using G*Power 3.1.9 indicated that a minimum sample size of 45 was required for paired t-tests (significance level α = 0.05, power = 0.95, effect size d = 0.50, one-sided test). Thus, the 142 participants in this study provided sufficient statistical power. This study was granted a separate exemption from the Institutional Review Board (IRB) of the author’s affiliated institution for the analysis of secondary data (IRB No. 2-1040709-AB-N-01-202105-HR-033-02).

### 2.2. Instruments

#### Patient Safety Communication Competency Tool

To evaluate patient safety communication competency, this study utilized an instrument originally developed by the author as part of a doctoral dissertation [19], which was based on the ‘effective communication for patient safety’ domain of the CPSI framework. The instrument comprises four core competencies (hereafter referred to as C1 to C4): C1 (Verbal and non-verbal communication), defined as showing effective communication skills to prevent adverse events; C2 (Communication in high-risk situations), defined as communicating effectively in specific high-risk situations to ensure patient safety; C3 (Written communication), defined as the accurate and effective use of written documentation; and C4 (Application of communication technologies), defined as utilizing communication technologies properly and effectively to ensure safe patient care.

During the tool development phase, the enabling competencies of the CPSI were adapted into 28 specific items covering knowledge, skills, and attitudes, tailored to the Korean nursing education context. Content validity was established by a panel of nine experts, including four nursing professors, one manager of a nursing education department, one manager of a quality assurance department, and three nurses in charge of nursing education at a tertiary hospital. The panel evaluated the appropriateness of each item for assessing patient safety communication competency and reviewed the accuracy of their classification into knowledge, skills, and attitude domains using a 4-point scale. The item-level Content Validity Index (CVI) ranged from 0.78 to 1.00. Based on the experts’ qualitative feedback, overlapping items were removed and terminology was refined, resulting in the final instrument.

In the present study, these 28 items were used to evaluate the dual aspects of perceived importance and confidence among new graduate nurses. Each item was rated on a 5-point Likert scale, ranging from 1 (Strongly disagree) to 5 (Strongly agree), with higher scores indicating greater degrees of perceived importance or confidence. In the original development study [19], the tool demonstrated high internal consistency, with Cronbach’s alpha coefficients ranging from 0.91 to 0.95.

Additionally, a structured questionnaire was used to collect data on the participants’ general characteristics. These included gender, age, satisfaction with their nursing major, satisfaction with clinical practice, grade point average (GPA), experience with patient safety education, and experience with patient safety communication education.

### 2.3. Data Analysis

Data were analyzed using SPSS for Windows, version 25.0 (IBM Corp., Armonk, NY, USA). The online survey was configured to require responses to all items before participants could proceed to the next page; therefore, no missing data were present among the 142 completed responses included in the analysis. No extreme outliers requiring exclusion were identified. The assumption of normality was evaluated using skewness and kurtosis; the absolute values for all measured variables were well within the acceptable limits (|skewness| < 3, |kurtosis| < 10) suggested by Kline [20], indicating no substantial violation of normality.

Descriptive statistics (frequencies, percentages, means, and standard deviations) were used to summarize participants’ general characteristics and their perceived importance and confidence scores. Differences between perceived importance and confidence levels were analyzed using paired t-tests. The correlation between perceived importance and confidence was examined using Pearson correlation coefficients. Statistical significance was set at *p* < 0.05.

Educational priorities for patient safety communication were identified using IPA. The four quadrants of the IPA matrix were divided using the overall mean scores of all 28 items as the cutoff values. Specifically, the crosshair for the matrix was set at 4.29 for perceived importance (*y*-axis) and 3.53 for confidence (*x*-axis). Scores above these cutoffs were classified as high, whereas those below the cutoff values were classified as low.

## 3. Results

### 3.1. General Characteristics of Participants

The total number of participants in this study was 142. The general characteristics of the participants are summarized in Table 1. Of the participants, 134 (94.4%) were female, with a mean age of 23.5 ± 2.5 years. At the time of the survey, participants’ clinical experience after graduation averaged 10.37 ± 15.14 days. The participants’ mean grade point average (GPA) in the nursing major was 3.64 ± 0.50 points out of 4.5. Regarding education, mean scores for satisfaction with the nursing major and clinical practice during their nursing education program were 3.80 ± 0.78 and 3.15 ± 0.96, respectively. 108 participants (76.1%) reported receiving patient safety education in their undergraduate curriculum, with a mean helpfulness score of 3.79 ± 0.83 out of 5 points. For patient safety communication specifically, 119 participants (83.8%) received education in their undergraduate curricula, which yielded a mean helpfulness score of 3.80 ± 0.85 out of 5.

### 3.2. Perceived Importance and Confidence in Patient Safety Communication

The mean scores for the participants’ perceived importance and confidence related to patient safety communication were 4.30 ± 0.56 and 3.55 ± 0.51, respectively (Table 2). Confidence scores were significantly lower than their perceived importance scores across all items (all *p* < 0.001). Regarding the perceived importance of patient safety communication, C1 had the highest score, followed by C2, C4, and C3. In terms of confidence, C1 had the highest confidence score, followed by C3, C4, and C2.

**Table 2:** Comparison of importance and confidence levels of patient safety communication (*N* = 142).

Competencies	Item	Importance	Confidence	Difference		t	*p*
		M ± SD		M ± SD	Ranking		
C1	-	4.37 ± 0.58	3.77 ± 0.52	0.60 ± 0.60	-	11.85	<0.001
	K1	4.23 ± 0.71	3.39 ± 0.80	0.85 ± 1.03	3	9.81	<0.001
	K2	4.21 ± 0.74	3.51 ± 0.85	0.70 ± 1.04	5	8.09	<0.001
	K3	4.54 ± 0.76	4.03 ± 0.81	0.51 ± 0.83	7	7.27	<0.001
	K4	4.23 ± 0.81	3.52 ± 0.84	0.71 ± 0.99	4	8.53	<0.001
	K5	4.27 ± 0.80	3.71 ± 0.81	0.56 ± 0.97	6	6.91	<0.001
	S1	4.46 ± 0.66	3.51 ± 0.88	0.96 ± 1.06	2	10.79	<0.001
	S2	4.41 ± 0.79	3.15 ± 1.00	1.26 ± 1.18	1	12.70	<0.001
	S3	4.42 ± 0.75	4.16 ± 0.91	0.26 ± 0.82	10	3.78	<0.001
	A1	4.54 ± 0.67	4.30 ± 0.67	0.23 ± 0.77	11	3.60	<0.001
	A2	4.38 ± 0.75	4.08 ± 0.79	0.30 ± 0.85	8	4.24	<0.001
	A3	4.42 ± 0.78	4.15 ± 0.82	0.27 ± 0.77	9	4.14	<0.001
C2	-	4.31 ± 0.57	3.36 ± 0.56	0.95 ± 0.72	-	15.78	<0.001
	K6	4.28 ± 0.79	3.77 ± 0.77	0.51 ± 0.95		6.35	<0.001
	K7	4.24 ± 0.72	3.33 ± 0.80	0.91 ± 1.09	7	9.92	<0.001
	K8	4.27 ± 0.80	3.23 ± 0.89	1.04 ± 1.17	3	10.65	<0.001
	K9	4.11 ± 0.80	3.09 ± 0.94	1.02 ± 1.11	4	10.92	<0.001
	S4	4.44 ± 0.70	3.48 ± 0.90	0.96 ± 1.07	6	10.70	<0.001
	S5	4.04 ± 0.85	2.86 ± 0.93	1.18 ± 1.15	2	12.23	<0.001
	S6	4.39 ± 0.74	3.05 ± 0.93	1.34 ± 1.14	1	13.97	<0.001
	S7	4.35 ± 0.72	3.34 ± 0.91	1.01 ± 1.14	5	10.53	<0.001
	A4	4.54 ± 0.63	4.11 ± 0.78	0.44 ± 0.84	8	6.21	<0.001
C3	-	4.18 ± 0.62	3.46 ± 0.63	0.72 ± 0.72	-	11.87	<0.001
	K10	3.85 ± 0.84	3.47 ± 0.91	0.37 ± 0.96	6	4.61	<0.001
	S8	4.48 ± 0.71	3.51 ± 0.89	0.96 ± 1.11	1	10.38	<0.001
	S9	4.35 ± 0.79	3.43 ± 0.89	0.92 ± 1.04	2	10.58	<0.001
	S10	4.13 ± 0.77	3.58 ± 0.84	0.54 ± 0.90	5	7.21	<0.001
	S11	4.16 ± 0.78	3.50 ± 0.84	0.66 ± 0.96	4	8.22	<0.001
	S12	4.14 ± 0.86	3.29 ± 0.93	0.85 ± 1.14	3	8.94	<0.001
C4	-	4.20 ± 0.69	3.42 ± 0.74	0.78 ± 0.86	-	10.84	<0.001
	K11	4.11 ± 0.81	3.49 ± 0.88	0.62 ± 1.00	2	7.37	<0.001
	S13	4.30 ± 0.76	3.36 ± 0.95	0.94 ± 1.11	1	10.09	<0.001

Ranking represents the magnitude of the difference between importance and confidence scores in descending order. C1, verbal and non-verbal communication; C2, communication in high-risk situations; C3, written communication; C4, application of communication technologies. C, competency; K, knowledge; S, skill; A, attitude; M, mean; SD, standard deviation. The complete descriptions of all survey items evaluated in this study are presented in Table 3.

**Table 3:** Classification of specific items into IPA quadrants for patient safety communication competencies (*N* = 142).

C	I. Keep up the Good Work	II. Concentrate Here	III. Low Priority	IV. Possible Overkill
C1	K3. Privacy and confidentiality S3. Active listening techniques A1. Respect and empathy A2. Patient-centered perspective A3. Cultural diversity.	S1. Explaining treatments and protocols S2. Structured team communication	K1. Health literacy needs K2. Importance of communication respecting cultural diversity K4. Communication for patient and family understanding	K5. Effect of communication on patient safety
C2	A4. Patient engagement for informed consent	S4. Communicating clinical urgency S6. Communication in high-risk situations (e.g., distress and conflict) S7. Safe communication in transitions of care	K7. Coordinating communication styles among team members K8. Informing patients and families of adverse events K9. Patient safety incident reporting system S5. Escalating concerns across authority gradients	K6. Importance of informing patients of discharge plans
C3	-	S8. Detailed and clear health record entries S9. Sufficient documentation for team comprehension	K10. Effect of abbreviations on patient safety S11. Use of official abbreviations and standardized guidelines S12. Documenting the rationale for deviations from guidelines	S10. Seeking and using well-designed patient education material
C4		S13. Structured approaches in technology (e.g., SBAR and read-back)	K11. Benefits, limitations, and professional responsibilities of technology	

C1, verbal and non-verbal communication; C2, communication in high-risk situations; C3, written communication; C4, application of communication technologies. C, competency; K, knowledge; S, skill; A, attitude. Each item is presented with its corresponding concise description; for example, K3 indicates “Privacy and confidentiality.” Shaded cells indicate items whose item-level IPA classifications were consistent with the competency-level IPA classification for each competency.

### 3.3. IPA of Patient Safety Communication

Based on the perceived importance and confidence scores, IPA was conducted at both the competency and item levels. At the competency level, the IPA results showed that C1 (verbal and non-verbal communication) was located in Quadrant I (Keep up the good work), indicating relatively high importance and high confidence. C2 (communication in high-risk situations) was positioned in Quadrant II (Concentrate here), characterized by relatively high importance but relatively low confidence. C3 (written communication) and C4 (application of communication technologies) were categorized in Quadrant III (Low priority), reflecting relatively low scores in both dimensions. No competencies were placed in Quadrant IV (Possible overkill).

**Figure 1:**
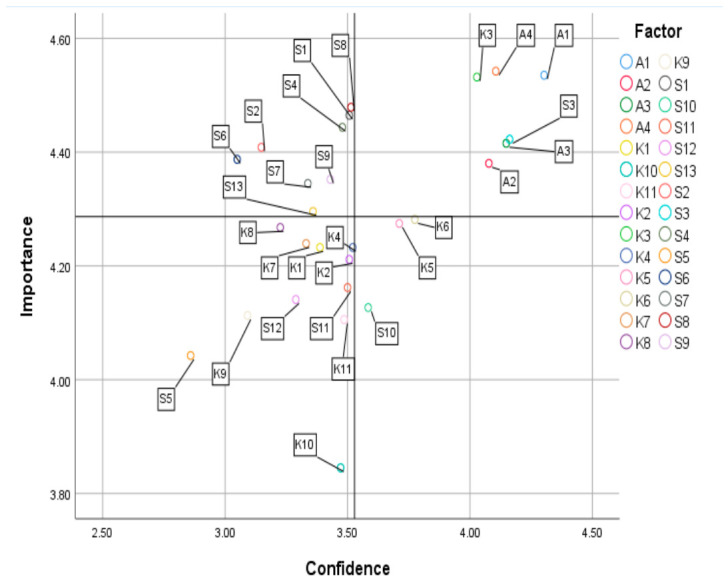
Importance-performance analysis of educational needs for patient safety communication among new graduates. The four quadrants indicate educational priorities: I (keep up the good work), II (concentrate here), III (low priority), and IV (possible overkill). K, knowledge; S, skills; A, attitude. The full description for each item code is provided in Table 3.

At the item level, the IPA results for knowledge (K), skill (S), and attitude (A) items showed variation within each competency, providing more detailed item-level priority information. In C1, several items, including K3, S3, and A1–A3, were classified in Quadrant I, consistent with the competency-level result. In C2, although the competency as a whole was located in Quadrant II, only selected items related to communicating clinical urgency, high-risk situations, and transitions of care, including S4, S6, and S7, were classified in the same quadrant. In C3 and C4, which were categorized as Quadrant III at the competency level, some skill-based items, including S8, S9, and S13, were located in Quadrant II. Finally, despite the absence of competencies in Quadrant IV, a few specific items, including K5, K6, and S10, were located in this quadrant, reflecting relatively high confidence but relatively lower perceived importance at the item level. The detailed item-level IPA results are presented in Figure 1 and Table 3.

## 4. Discussion

This study assessed perceived importance and confidence levels related to patient safety communication competencies among new graduate nurses using IPA to inform future educational strategies for undergraduate and clinical nursing programs. Based on the four communication competencies derived from the CPSI framework, the IPA results showed distinct priority patterns across competency and item levels.

Notably, C1 (verbal and non-verbal communication) emerged in Quadrant I (Keep up the good work), reflecting both high perceived importance and high confidence among new graduate nurses (importance: 4.37, confidence: 3.77). Collectively, it is noteworthy that this quadrant predominantly comprised foundational interpersonal skills, such as active listening, empathy, and patient-centeredness (shaded items in Table 3). Because therapeutic communication has long been emphasized as a core value in nursing guidelines [6,11] and actively integrated into nursing education [21,22], the placement of C1 in Quadrant I may reflect the continued influence of these educational efforts. Therefore, current pedagogical approaches for these fundamental communication skills should be maintained and consistently reinforced.

At the item level, however, several practical skill items were located in Quadrant II (Concentrate here), indicating areas where additional educational attention may be needed. Within C1, for instance, these items included the clear explanation of treatments and protocols to patients (S1; importance: 4.46, confidence: 3.51) and structured information transfer to team members (S2; importance: 4.41, confidence: 3.15). Regarding patient interaction, nursing students should understand that patients with low health literacy often face barriers to participating in the care process or communicating with healthcare providers [23], and these barriers can threaten health outcomes such as medication safety [24]. The COVID-19 pandemic and its aftermath have highlighted the vulnerability of clinical practice opportunities [25], including direct patient interactions, which may limit students’ hands-on experience in addressing patient communication barriers. To address this issue, nursing curricula could incorporate simulations using standardized patients that encourage active verbalization. Rather than passively learning the concept of health literacy, students may benefit from repeated practice in tailored communication strategies—such as the “teach-back” method and “Ask Me” prompts [23]. This experiential training may help translate theoretical knowledge into the practical confidence needed to effectively assess and confirm patient comprehension.

Beyond patient-facing interactions, the Quadrant II results also point to a need for further strengthening team-based patient safety communication, specifically regarding structured information transfer. While it is widely established that communication failures during care transitions—such as handovers—account for a majority of sentinel events [2], the findings of this study suggest that new graduate nurses may feel insufficiently prepared to execute these high-stakes exchanges. Given that handovers carry a high risk of information distortion or omission that can rapidly precipitate medical errors [26], the low confidence observed in this area supports the need to move beyond theoretical instruction toward practice-oriented training.

To address this vulnerability, standardized methods such as SBAR and I PASS the BATON should be embedded as core practical competencies rather than mere theoretical mnemonics [2,27]. The primary value of these tools lies in creating a shared mental model between senders and receivers, ensuring predictable information delivery and preventing the cognitive overload that delays patient care [2]. However, communication education [21,28] and patient safety education [29] in Korean nursing curricula tend to be predominantly theoretical in both content and method, often falling short of meeting students’ educational needs for practical, hands-on experience in patient safety communication. To bridge this gap, the recent literature suggests that theoretical patient safety education may be strengthened through participatory learning approaches, such as practical training in hospital settings, simulation-based training, and virtual simulation training [30]. Moreover, by establishing standardized communication protocols as best practices within the curriculum and fostering close academic–clinical partnerships with hospitals, institutions can provide nursing students with seamless opportunities to actively practice structured handovers in real-world settings, facilitating the translation of classroom theory into clinical confidence.

Another important area requiring educational attention is C2 (communication in high-risk situations). Although participants ranked this domain as the second most important, it yielded the lowest overall confidence scores (importance: 4.31, confidence: 3.36). Collectively, the IPA results reveal a notable discrepancy: while participants reported relatively higher confidence in predictable scenarios—such as obtaining informed consent (Quadrant I)—they reported lower confidence when navigating unpredictable, high-stakes environments (Quadrant II). Specifically, communicating clinical urgency (S4; importance: 4.44, confidence: 3.48), ensuring safe communication under distressing or conflicting conditions (S6; importance: 4.39, confidence: 3.05), and maintaining safety in transitions of care (S7; importance: 4.35, confidence: 3.34) emerged as areas requiring educational attention. This gap suggests that while new graduates understand the imperative of safety in high-risk situations, they question their practical confidence to execute it under pressure.

To prepare students for these high-pressure scenarios, nursing curricula should incorporate specific teamwork strategies. During clinical crises, for instance, students should be trained to take a moment to pre-brief and examine progress, required tools, possible complications, and expected outcomes together [31]. In addition, they should practice call-out techniques—stating critical patient information aloud—to simultaneously share updates and help the team anticipate next steps [32]. Beyond acute clinical crises, interpersonal conflicts and emotional distress can also severely disrupt information flow and threaten patient safety [32,33]. Therefore, educational efforts should incorporate scenarios where conflict hinders effective communication. Through practical examples, students need opportunities to practice structured techniques in simulated high-risk situations, such as the DESC method—a framework for resolving conflicts by objectively describing the situation, expressing concerns, suggesting alternatives, and stating safety-focused consequences as the common team goal [32].

However, simply memorizing these acronyms is insufficient. Because high-stress environments often trigger psychological barriers that induce silence [13], students can benefit from engaging in high-fidelity simulations where they are encouraged to practice active verbalization. By repeatedly vocalizing their concerns using frameworks like DESC and call-outs in simulated crises, students can facilitate the translation of theoretical knowledge into the practical communication skills required to successfully advocate for patients in real-world clinical settings. In this context, virtual simulations offer a realistic, risk-free environment where students can repeatedly practice these skills, which may help support their practical confidence and potentially reduce the psychological burden often associated with traditional in-person training [34]. A recent meta-analysis examining the effects of virtual simulation on clinical reasoning among nursing students and nurses also suggested that experiential simulation-based learning can improve applied knowledge and clinical performance, particularly when education incorporates multiple patient-management scenarios and postscenario feedback [35]. These findings support the value of repeated, scenario-based practice in strengthening patient safety communication competencies.

Finally, of the four competencies, C3 (written communication) and C4 (application of communication technologies) were perceived as relatively lower-priority domains overall. For C3, participants reported the lowest perceived importance (mean: 4.18) but the second-highest confidence (mean: 3.46). This may indicate a potential under-recognition of the safety relevance of documentation, possibly because participants felt relatively proficient in basic data entry tasks. Nevertheless, the IPA results highlighted specific documentation tasks—namely, maintaining appropriately detailed electronic health records (EHRs) and comprehensive patient histories and care plans—as high-priority areas requiring attention.

Written communication complements verbal interaction by providing verifiable, detailed information [2]. In modern healthcare, information systems such as electronic health records (EHRs) are essential for this process, allowing patient data to be shared systematically and in real-time [36,37]. This real-time documentation enables health care providers to communicate securely and build a shared mental model, ultimately improving care quality and safety [2]. Within this digitized environment, nurses are responsible for maintaining accurate and complete records throughout the entire nursing process—from data collection and diagnosis to planning, implementation, and evaluation—using critical thinking [6]. Because any delays in recording can severely hinder information sharing and precipitate treatment delays or medical errors [2], education on safe record-keeping should be prioritized in undergraduate curricula. Since most hospital records are digitized, students should be explicitly trained to directly enter patient data using computers and carefully verify the completeness of the information.

Similarly, although C4 (application of communication technologies) was categorized as a relatively lower-priority domain overall (importance: 4.20, confidence: 3.42), the item-level IPA indicated that using structured approaches in technology-mediated communication emerged as an area requiring educational attention (S13; importance: 4.30, confidence: 3.36). While face-to-face communication remains the most effective method for healthcare teams [2], temporal and situational constraints often necessitate the use of technology-mediated communication in clinical practice. Although the integration of health information technology inherently reduces certain safety incidents through real-time data sharing [36,37,38], remote communication can introduce new avenues for error. To counteract this, implementing structured communication frameworks may serve as an important safety barrier. For instance, utilizing SBAR during telephone interactions between nurses and physicians has been shown to positively impact patient outcomes by decreasing mortality and readmission rates [39]. Furthermore, employing “read-back” and “check-back” methods is crucial for reconfirming information, as these closed-loop techniques are proven to detect critical errors [40] and enhance the clarity of information delivery [41]. Therefore, undergraduate nursing programs should educate students on these guidelines and techniques to ensure safe and effective communication when using communication technologies.

Although no overall competencies were placed in Quadrant IV (Possible overkill), several item-level results related to the effects of communication and the use of educational materials (e.g., K5, K6, S10) were located in this quadrant. These findings should be interpreted cautiously. Rather than indicating that education in these areas is unnecessary, this pattern may suggest that new graduate nurses perceived these topics as relatively less important despite reporting relatively high confidence. Therefore, future education may need to help learners more explicitly connect the effects of communication, discharge-plan communication, and the use of patient education materials with concrete patient safety outcomes, rather than addressing them only as isolated concepts.

Beyond undergraduate education, the findings of this study provide meaningful insights for clinical practice, continuing education, and organizational support. In clinical settings, orientation programs for new graduate nurses could be tailored to address the confidence gaps identified in this study, particularly in communication during high-risk situations involving clinical urgency, emotional distress and conflict, and care transitions. Furthermore, continuing education programs should periodically assess these specific patient safety communication competencies and provide targeted supplemental training to support the ongoing development of these critical skills throughout nurses’ careers. At the organizational level, patient safety communication in everyday clinical practice should be supported through the dissemination of comprehensive guidelines and standardized communication protocols accompanied by practical examples.

## 5. Limitations

This study has several limitations. First, the data were collected in 2020 from new graduates at four nursing colleges in Korea, during the rapid spread of the COVID-19 pandemic; therefore, the findings should be generalized with caution. Although the participants had already graduated and were transitioning into clinical practice, making them less likely to be directly affected by changes in undergraduate educational environments, the heightened social and healthcare crisis awareness during the pandemic may have influenced their perceptions of the importance of patient safety communication and their self-reported confidence levels. In addition, given the voluntary online survey design, 40.6% of those who initiated the survey did not complete it, which may have introduced response bias. Together with the convenience sampling approach, this noncompletion rate may limit the representativeness of the sample and should be considered when interpreting the findings.

Second, confidence related to patient safety communication was evaluated using a self-reported questionnaire; therefore, participants’ responses may not fully reflect their actual clinical competence. Future studies should consider evaluating actual communication performance through objective methods, such as observing simulated scenarios. In addition, future research should explore demographic and educational factors associated with patient safety communication competencies using study designs appropriate for identifying predictors, including regression analysis, and develop intervention studies aimed at enhancing these essential skills among new graduate nurses.

## 6. Conclusions

In conclusion, this study identified communication in high-risk situations as a key educational priority for patient safety communication among new graduate nurses. To address the confidence gap observed in this area, undergraduate curricula may incorporate scenario-based learning, including virtual simulations, in which communication directly affects patient safety. Such experiential approaches may help nursing students better understand high-risk scenarios and practically apply specific communication skills to support safe care. Targeted educational strategies are also needed to address written communication and the use of communication technologies, particularly to emphasize their relevance to preventing communication-related errors. By using IPA to identify specific communication vulnerabilities, this study provides empirically derived priorities and practical suggestions for strengthening patient safety communication education. These findings may support the development of educational strategies that help new nurses communicate more safely and confidently during the transition to clinical practice.

## Figures and Tables

**Table 1:** General characteristics of the participants (*N* = 142).

Variables	Categories	*n* (%)	M ± SD
Gender	Male	8 (5.6)	
	Female	134 (94.4)	
Age (years)			23.51 ± 2.52
Clinical experience (days)			10.37 ± 15.14
GPA (out of 4.5)			3.64 ± 0.50
Satisfaction with nursing major			3.80 ± 0.78
Satisfaction with clinical practice			3.15 ± 0.96
Patient safety education	Not received	34 (23.9)	
	Received	108 (76.1)	
Helpfulness of patient safety education †			3.79 ± 0.83
Patient safety communication education	Not received	23 (16.2)	
	Received	119 (83.8)	
Helpfulness of patient safety communication education †			3.80 ± 0.85

† Among participants who received the education.

## Data Availability

The data presented in this study are available upon request from the corresponding author. The reason for this restriction is that the data are not publicly available due to privacy and ethical restrictions, as the original informed consent provided to participants guaranteed that their data would be managed confidentially and used only for research purposes.

## Abbreviations

The following abbreviations are used in this manuscript:

C	Competency
CPSI	Canadian Patient Safety Institute
EHRs	Electronic Health Records
IPA	Importance-Performance Analysis
SBAR	Situation-Background-Assessment-Recommendation
